# Bridging Terrestrial Water Storage Anomaly During GRACE/GRACE-FO Gap Using SSA Method: A Case Study in China

**DOI:** 10.3390/s19194144

**Published:** 2019-09-24

**Authors:** Wanqiu Li, Wei Wang, Chuanyin Zhang, Hanjiang Wen, Yulong Zhong, Yu Zhu, Zhen Li

**Affiliations:** 1 Chinese Academy of Surveying & Mapping, Beijing 100830, China; 2 School of Geography and Information Engineering, China University of Geosciences (Wuhan), Wuhan 430074, China; 3 Institute of International Rivers and Eco-Security, Yunnan University, Kunming 650500, China; 4 GNSS Research Center, Wuhan University, Wuhan 430079, China

**Keywords:** GRACE, TWSA, SSA, prediction, data gap

## Abstract

The terrestrial water storage anomaly (TWSA) gap between the Gravity Recovery and Climate Experiment (GRACE) and its follow-on mission (GRACE-FO) is now a significant issue for scientific research in high-resolution time-variable gravity fields. This paper proposes the use of singular spectrum analysis (SSA) to predict the TWSA derived from GRACE. We designed a case study in six regions in China (North China Plain (NCP), Southwest China (SWC), Three-River Headwaters Region (TRHR), Tianshan Mountains Region (TSMR), Heihe River Basin (HRB), and Lishui and Wenzhou area (LSWZ)) using GRACE RL06 data from January 2003 to August 2016 for inversion, which were compared with Center for Space Research (CSR), Helmholtz-Centre Potsdam-German Research Centre for Geosciences (GFZ), Jet Propulsion Laboratory (JPL)’s Mascon (Mass Concentration) RL05, and JPL’s Mascon RL06. We evaluated the accuracy of SSA prediction on different temporal scales based on the correlation coefficient (*R*), Nash–Sutcliffe efficiency (NSE), and root mean square error (RMSE), which were compared with that of an auto-regressive and moving average (ARMA) model. The TWSA from September 2016 to May 2019 were predicted using SSA, which was verified using Mascon RL06, the Global Land Data Assimilation System model, and GRACE-FO results. The results show that: (1) TWSA derived from GRACE agreed well with Mascon in most regions, with the highest consistency with Mascon RL06 and (2) prediction accuracy of GRACE in TRHR and SWC was higher. SSA reconstruction improved *R*, NSE, and RMSE compared with those of ARMA. The *R* values for predicting TWS in the six regions using the SSA method were 0.34–0.98, which was better than those for ARMA (0.26–0.97), and the RMSE values were 0.03–5.55 cm, which were better than the 2.29–5.11 cm RMSE for ARMA as a whole. (3) The SSA method produced better predictions for obvious periodic and trending characteristics in the TWSA in most regions, whereas the detailed signal could not be effectively predicted. (4) The predicted TWSA from September 2016 to May 2019 were basically consistent with Global Land Data Assimilation System (GLDAS) results, and the predicted TWSA during June 2018 to May 2019 agreed well with GRACE-FO results. The research method in this paper provides a reference for bridging the gap in the TWSA between GRACE and GRACE-FO.

## 1. Introduction

Terrestrial water storage (TWS) is an important aspect of the water cycle. Accurate estimates and reliable predictions of TWS anomaly (TWSA) are crucial for understanding global and local water cycle processes, for researching and forecasting climate change, agriculture production adjustment, and preventing natural disasters [1]. Remote sensing and hydrological station monitoring both have limitations, which pose challenges for effectively monitoring TWSA.

Since 2002, Gravity Recovery and Climate Experiment (GRACE) has achieved success in monitoring changes in the mass on the Earth’s surface [2,3], especially in TWSA research [4,5,6,7,8,9,10,11]. As the GRACE mission ended in after 15 years October 2017, the GRACE Follow-On (GRACE-FO) mission gravity satellite was launched in May 2018. The satellite will continue to monitor changes in the global gravity field and is expected to be applied in most fields of hydrology and geodesy. GRACE’s Release Level 06 (RL06) geoid spherical harmonic model (GSM) from the Center for Space Research (CSR) was updated in June 2017, the latest GRACE-FO RL06 GSM data were released recently, and its data period ranges from June 2018 to May 2019. However, GRACE data retrived after August 2016 are not available. Therefore, the resulting gap between these two missions is about 20 months. The data gap between two generations of gravity satellites has resulted in gravity satellites failing to provide continuous TWSA information. Therefore, a method is needed to effectively predict GRACE TWSA signals, which would be beneficial for the sustainable management of water resources.

Most scholars are using statistical and machine learning methods, such as the least squares method (LSM) nonlinear regression, and correlation analysis [12,13,14]; time series models, such as auto-regressive and moving average model (ARMA), auto-regressive integrated moving average model (ARIMA), and seasonal auto-regressive integrated moving average (SARIMA) [15,16,17]; artificial neural networks (ANNs) [16,18], wavelet-artificial neural networks (WA-ANNs) [19,20], support vector regression (SVR) [21], and other methods, to evaluate their performance from the perspective of predicting groundwater changes. The first few methods are simple to implement, and the function of water storage changes with time is constructed according to the least squares principle. However, the least squares parameter estimation is susceptible to abnormal signals in the time series, which leads to limited prediction accuracy. In contrast, the prediction accuracies of ANN, WA-ANN, and SVR algorithms are higher, but the implementation processes are complex and the prediction accuracies still depend on a large amount of meteorological data, which is a physical parameter constraint. Therefore, to balance computational efficiency and prediction accuracy, we adopt a new prediction method, the singular spectrum analysis (SSA) iterative prediction algorithm.

In contrast to the above methods, SSA is derived from the Karhunen–Loeve decomposition theory, which is a principle component analysis method for analyzing one-dimensional time series [22]. The SSA method has obvious advantages: Unrestricted sine wave assumption, no prior information is required, stable identification, and an enhanced periodic signal. In the dynamic reconstruction of a time series of finite length, combined with the empirical orthogonal function, SSA can extract as much useful information as possible from a noise-containing time series. This method has obvious advantages and its application has gradually increased [23,24,25]. However, SSA has rarely been applied to the prediction of GRACE TWSA during its data gap. 

In this paper, we propose the adoption of SSA for the prediction of TWSA during the data gap between the GRACE and GRACE-FO inversion results.The experiments were conducted in the North China Plain (NCP), Southwest China (SWC), Three-River Headwaters Region (TRHR), Tianshan Mountains Region (TSMR), Heihe River Basin (HRB), and Lishui and Wenzhou area (LSWZ). Combined with the Mascon products from Center for Space Research (CSR), Helmholtz-Centre Potsdam-German Research Centre for Geosciences (GFZ), Jet Propulsion Laboratory (JPL), and the Global Land Data Assimilation System (GLDAS) hydrological models, we analyze the performance of SSA in predicting regional TWSA in terms of the inversion result reliability verification, construction of SSA predictive models, and SSA prediction method accuracy assessment. The ARMA model was compared with our method on different time scales to explore the effectiveness and advantages of the SSA method for use in predicting the gap in GRACE TWSA.

## 2. Data

### 2.1. GRACE RL06 Spherical Harmonic Data

The spherical harmonic data for GRACE level-2 Release Level 06 (RL06) from the CSR (Center for Space Research, University of Texas, Austin, TX, USA) (http://www2.csr.utexas.edu/grace/) were used in this study. The period of data ranged from January 2003 to August 2016. Similar to the RL05 data, the data are regularized spherical harmonic coefficients, deducting solid tides, ocean tides, non-tidal atmospheric and ocean signals, and associated gravity disturbances. Compared with RL05, RL06 data has been optimized and improved in terms of parameter selection, algorithm design, data editing, and background field model construction; its accuracy has also been improved [26]. The C_20_, C_10_, S_10_, and S_11_ in the gravity field model were replaced by the second-order term from Satellite Laser Ranging (SLR) [27] and the first-order term from Swenson [28].

### 2.2. GRACE Mascon Solution

We used Mascon RL05- and RL06-version data for verification analysis. The RL05 version data were released by CSR, GFZ, and JPL. The RL06 Mascon data that were available for download at the time were only provided by JPL. The resolution of Mascon RL05 data is 1° × 1° and the period ranged from January 2003 to January 2017; the resolution of Mascon RL06 data is 0.5° × 0.5° and the period ranged from January 2003 to June 2017. The leakage error has been processed in the Mascon, no filtering was required [29], its C_20_ was replaced by SLR [30], and the first-order term was corrected with glacial isostatic adjustment (GIA) [31]. Compared with the GRACE geoid spherical harmonic model (GSM) products, Mascon results were more accurate and widely used in the verification analysis of GRACE inversion results [32,33].

### 2.3. GLDAS Hydrological Model

The GLDAS global hydrological model was established by NASA’s Goddard Space Flight Center and the US National Environmental Forecast Center [34] and reflected the changes of soil water, ice, and snow on land surface (https://mirador.gsfc.nasa.gov/). The spatial resolution was 0.25° × 0.25° and the monthly value included four land surface models (e.g., Noah, VIC, CLM, and MOSAIC). Due to the limited data download access in the early stage of our study, model data from January 2003 to May 2019 were collected. We selected the Noah soil water model for comparison and analysis during this period. 

## 3. Method

### 3.1. GRACE Inversion Method

A South–North stripe existed in the GRACE gravity field model. The stripe contained high-order noise and related errors. To reduce the stripe phenomenon, we used a 300 km fan filter [35,36] and a P3M15 decorrelation filter [37]. The inversion model was further used to obtain the TWSA in the six study regions. The combined filtering inversion model was as follows:

(1) $$\Delta h_{w}(\theta ,\lambda )=\frac{R\rho_{e}}{3\rho_{w}}\sum_{n=0}^{N}W_{n}\sum_{m=0}^{n}\frac{2n+1}{1+{k^{\prime }}_{n}}[\Delta C_{nm}\cos m\lambda +\Delta S_{nm}\sin m\lambda ]\cdot W_{m}\cdot {\bar{P}}_{nm}(\cos\theta )$$

where Δ*h_w_* is the surface mass change expressed by equivalent water height change; *θ* and *λ* are the colatitude and longitude of the calculation point, respectively; $\left(\Delta C_{nm},\Delta S_{nm}\right)$ is the change in normalized SHCs with degree *n* and order *m*; ${\bar{P}}_{nm}(\cdot )$ is the fully normalized associated Legendre function with degree *n* and order *m*; $k_{n}^{\prime }$ is the load love number of degree *n*; $\rho_{e}\approx 5.5\times 10^{3}kg/m^{3}$ is the average density of solid earth; *R* is the average radius of the earth; and $W_{n}$ and $W_{m}$ are the degree-dependent and order-dependent smoothing functions, respectively.

### 3.2. SSA Principle

SSA is a time series processing method for principal component analysis. With this method, for a one-dimensional time series after being centralized, a time-delay matrix was constructed according to the given embedded space dimension, a certain number of principal components were truncated, and the trend and periodic term signals were reconstructed [22]. In general, a window length of *M* can better recognize the oscillation between *M*/5 and *M*. For the time series of the known cycle, to better separate the periodic part, the window length was usually taken as a multiple of the period.

For the monthly time series derived from GRACE $\left\{x_{1},x_{2},\cdots ,x_{N}\right\}$, the embedded dimension was *M* and the time lag matrix $M\times \left(N-M+1\right)$ was constructed, which was the space coordinate matrix X:

(2) $$X=\left[\begin{array}{cccccc} x_{1} & x_{2} & \cdots & x_{i+1} & \cdots & x_{N-M+1}\\ x_{2} & x_{3} & \cdots & x_{i+2} & \cdots & x_{N-M+2}\\ \vdots & \vdots & \vdots & \vdots & \vdots & \vdots \\ x_{M} & x_{1+M} & \cdots & x_{i+M} & \cdots & x_{N} \end{array}\right]=\left[\begin{array}{cccccc} X_{1} & X_{2} & \cdots & X_{i+1} & \cdots & X_{N-M+1} \end{array}\right]$$

(1) Calculate the M×N auto-covariance matrix $T_{x}$ of X.

The singular value decomposition of the lag-covariance matrix generated a set of eigenvalues $\lambda_{k}$ and a matrix of eigenvectors $E^{k}$ (but plural) of rank equal to the embedding dimension.

(3) $$T_{x}=\left[\begin{array}{cccccc} c(0) & c(1) & \cdots & \cdots & \cdots & c(M-1)\\ c(1) & c(0) & c(1) & \cdots & \cdots & \cdots \\ \cdots & c(1) & \cdots & \cdots & \cdots & \cdots \\ \vdots & \vdots & \vdots & \vdots & \vdots & \vdots \\ \cdots & \cdots & \cdots & \cdots & \cdots & c(1)\\ c(M-1) & \cdots & \cdots & \cdots & c(1) & c(0) \end{array}\right]$$

where $T_{x}$ is a Toeplitz matrix and c(j) is the unbiased auto covariance function of $x_{i}$ with time delay *j*. The relationship between c(j) and $x_{i}$ is:

(4) $$c(j)=\frac{1}{N-j}\sum_{t=1}^{N-j}x_{t}x_{t+j}\;(0\leq j\leq M-1)$$

The $T_{x}$ matrix was decomposed into M eigenvalues,$\lambda_{k}(k=1,2,\cdots ,M)$, which was ordered from large to small, $\lambda_{1}>\lambda_{2}>\cdots >\lambda_{M}\geq 0$, and the corresponding eigenvector $E^{k}$ was solved.

(2) Calculate the projection of vector $X_{i}$ on $E^{k}$. 

The principal component (PC) $a_{i}^{k}$ can be expressed as the projection of the $X_{i}$ state vector on $E^{k}$, which is as follows:

(5) $$a_{i}^{k}=X_{i}\cdot E^{k}=\sum_{j=1}^{M}x_{i+j}E_{j}^{k}\;(0\leq i\leq N-M)$$

(3) Reconstruct the component. 

According to the projection $a_{i}^{k}$ and eigenvector $E^{k}$, every reconstruction time series of the PC can be expressed as:

(6) $$x_{i}^{k}=\left\{\begin{array}{cc} \frac{1}{i}\sum_{j=1}^{i}a_{i-j}^{k}E_{j}^{k} & \left(1\leq i\leq M-1\right)\\ \frac{1}{M}\sum_{j=1}^{M}a_{i-j}^{k}E_{j}^{k} & \left(M\leq i\leq N-M+1\right)\\ \frac{1}{N-i+1}\sum_{j=i-N+M}^{M}a_{i-j}^{k}E_{j}^{k} & \left(N-M+2\leq i\leq N\right) \end{array}\right.$$

(4) Reconstruct the time series.

In Equation (6), there are three forms of the reconstructions due to the edge effects in the Vautard et al. approach. The PC can reflect the variation characteristics of the time series, which determined the appropriate truncation order of PC *p*, and completing signal reconstruction to achieve the extraction of the main components of the GRACE signal:

(7) $${\hat{x}}_{i}=\sum_{k=1}^{p}x_{i}^{k}\;(i=1,2,\cdots ,N)$$

### 3.3. SSA Iterative Prediction Method

In this study, we considered that there were missing data in the GRACE prediction period, and the SSA method was proposed to predict the missing GRACE signal. The pre-GRACE signal in the study area was used as the training sample to test for optimal window width. The reconstructed components (RC) period pair was identified according to the W-correlation method and the paired plural eigenvalues [38]. On this basis, the fast Fourier transform (FFT) period was used to improve the reconstruction accuracy. The GRACE signal was predicted by truncating the order k and the window width *w*, and the latter signal was used as a test to verify the prediction performance of the SSA. The specific steps were:

(1) Set the prediction length *n* and add *n* zero values to the end of the original sequence (of length *N*) to construct a new GRACE sequence with total length *N* + *n*.
(2) Perform SSA decomposition on the new sequence of length *N* + *n* constructed in step (1), select *n* values at the end of the first principal component (PC1) as the predicted value, replace the original sequence, construct a new sequence, and repeat the process. The convergent condition is that the root mean square error (RMSE) of the difference between the two decomposed PC1s is less than the threshold. The threshold is determined empirically, and is generally taken as 0.01 mm [39,40,41].
(3) At the end of the loop process in step (2), the new sequence generated is used for the starting data in step (3), and the SSA decomposition is performed again. A new sequence is constructed by superimposing the *n* predicted values at the end of PC1 and PC2, and the process is repeated until the PC1 + PC2 sequence converges.
(4) Repeat the above process until the same component obtained by the previous SSA decomposition is replaced by the sum of k principal components (PCs). The series of length *n* at the end of these PCs is the main PC prediction series of GRACE (denoted by PCP).

**Figure 1:**
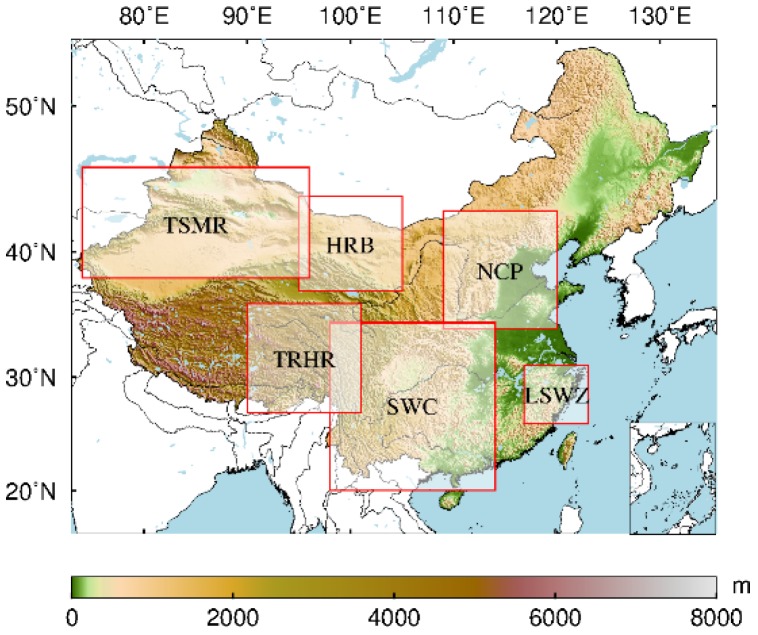
Overview map of the study area in China.

Some scholars have conducted research on GRACE TWSA in some representative regions of China [8,42,43,44,45,46,47,48]. For this reason, we also selected the following regions as experimental areas: North China Plain (NCP) (109°–120° E, 34°–43° N), Southwest China (SWC) (95°–115° E, 20°–35° N), Three-River Headwaters Region (TRHR) (90°–101° E, 27°–36° N), Tianshan Mountains Region (TSMR) (74°–96° E, 38°–46° N), Heihe River Basin (HRB) (95°–105° E, 37°–44° N), and Lishui and Wenzhou area (LSWZ) (117°–123° E, 26°–31° N). The SSA method was used to predict and analyze the TWSA in the six regions derived from GRACE. The location map of the study area is shown in Figure 1.

## 4. Results and Analysis

### 4.1. Comparison of GRACE Results with Mascon

Filtering suppressed noise, but also caused destruction of the signal amplitude and attenuation of the spatial distribution, which is called the leakage effect. To restore relatively realistic surface TWS changes, correcting the GRACE leakage error was necessary. In this study, we adopted scale factors to correct the leakage error and restore amplitude, which were calculated using a single-scale factor method based on the GLDAS model, as shown in Table 1.

Table 1 shows that differences occurred in the filtering scale factors for different regions, and the attenuation signal was further restored according to the above scale factors. In this study, we calculated the regional TWSA from January 2003 to August 2016 and compared them with Mascon RL05 data from JPL, CSR, and GFZ, and Mascon RL06 data from JPL, and then we evaluated their accuracies.

The results of JPL Mascon RL05 produced mutations in the TSMR and the HRB areas in partial time periods. The data in this period possibly had gross errors; therefore, the data during this time period were not considered. To improve clarity, the vertical axis range was adjusted to the normal data range.

**Figure 2:**
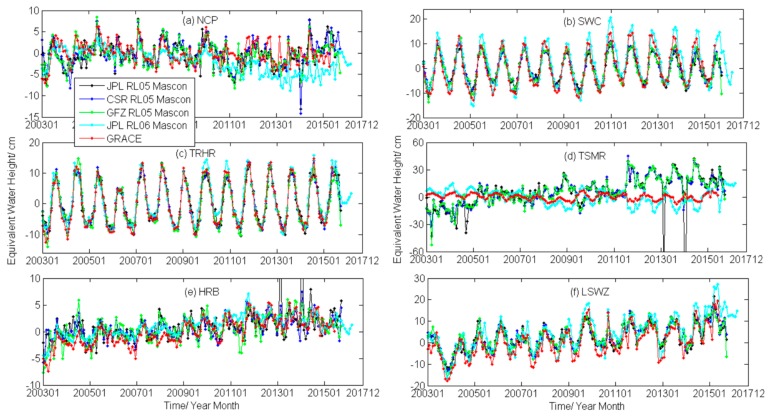
The terrestrial water storage anomaly (TWSA) derived from Gravity Recovery and Climate Experiment (GRACE) in typical regions in China compared with Mascon.

Figure 2d shows that the TSMR results derived from GRACE were more consistent with Mascon RL06, and the accuracy relative to each Mascon of the inversion results in the TSMR area is shown in Table 2. In addition to the large difference during the latter period in the NCP, the GRACE inversion results agreed well with the four Mascon products in the overall trend. The TWS change in the NCP, derived from GRACE, was in good agreement with the Mascon RL06 model from 2003–2011, but the accuracy of the GRACE results relative to RL06 was low after 2011, which may be related to the solution error of the RL06 Mascon model after 2011. The temporal fluctuation in TWS in the TSMR was consistent with the RL06 solution, but was negatively correlated with the RL05 solution. This signal difference may be due to the inconsistent algorithm used for solving the observation data when producing RL06 and RL05 Mascon model solutions.

To quantitatively evaluate the degree of agreement between GRACE spherical harmonic and Mascon results, we calculated the correlation coefficient (*R*) and the proximity Nash–Sutcliffe efficiency (NSE) of five regions, omitting TSMR, as shown in Table 2.

Table 2 shows that the *R* value between TWSA derived from GRACE in the five regions and the Mascon RL06 results was the highest and the NSE was the largest. The results produced from Mascon RL06 effectively verified the GRACE inversion results presented here. In the following experiment, the Mascon RL06 results were selected for predictive analysis verification.

### 4.2. SSA Forecasting Capability Verification Analysis

#### 4.2.1. Weighted Correlation Analysis and Signal Reconstruction

Due to space limitations, we chose the results with obvious periodic characteristics from the SWC region derived from GRACE as an example to illustrate the process of periodic pair identification and signal reconstruction performed by SSA before prediction. According to our experience, a 12-month periodic signal usually existed in the GRACE results. The SSA prediction could be implemented in MATLAB; the basic SSA code is available in a Github repository (https://github.com/1404855692lwq/lwqwhm/blob/master/SSA_basic_code.m). When SSA PC decomposition was performed, window width was designed for multiples of the known period. The window width *w* was continuously tested to achieve periodic separation and PC decomposition. The optimum window width *w* of 60 was determined by testing.

**Figure 3:**
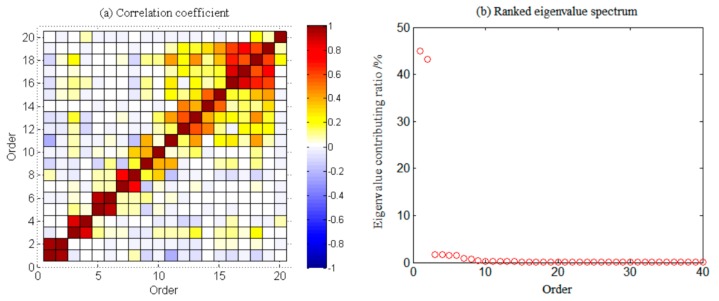
W-correlation analysis of TWSA in Southwest China.

**Figure 4:**
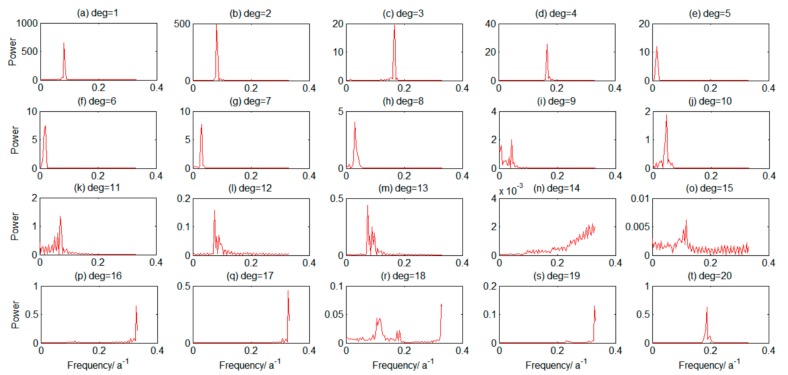
Fourier transform (FFT) period detection results of TWSA in Southwest China.

Weighted correlation analysis was used to quantitatively describe the correlation between each PC, PCs with the same periodic signal characteristics were grouped, which is shown in Figure 3, and the portion with a higher correlation coefficient was reconstructed. To identify the periodic characteristics of each PC, periodic detection was performed by FFT, and the spectrum diagram is shown in Figure 4.

Figure 4 shows that each two adjacent orders in the first eight order terms were period pairs (e.g., the first-order and second-order, the third-order and fourth-order PCs, etc., had the same period), and the signal intensities were relatively close. In Figure 3 and Figure 4, only the first 20 orders are shown to clarify the correlation coefficient presentation between each principal component more clearly. It is also possible to select the 30th and 40th order. According to experience, the time series periodic signals extracted by SSA were generally distributed in the first 20 orders. Therefore, we presented results for the first 20 reconstructed components in Figure 3 [40]. Figure 3 shows that the PC correlation coefficients of each group in the first eight orders were higher than 0.76, the contributing rate of the first eighth-order eigenvalues was 96.21%. Combining Figure 3 and Figure 4 explains that the first eighth-order PC was the periodic component, and the first eight orders were selected for signal reconstruction.

Based on the previous idea of SSA signal decomposition and reconstruction, we conducted the subsequent SSA signal prediction experiment.

#### 4.2.2. Comparison of Predicted Signals with True Values

The GRACE time series data from January 2003 to December 2012 (120 months in total) were used as training samples to perform SSA signal decomposition and reconstruction. The later data from January 2013 to August 2016 (44 months) were used as a true value test to assess the accuracy of the SSA method on short, mid-short, medium, and long-term time scales.

**Figure 5:**
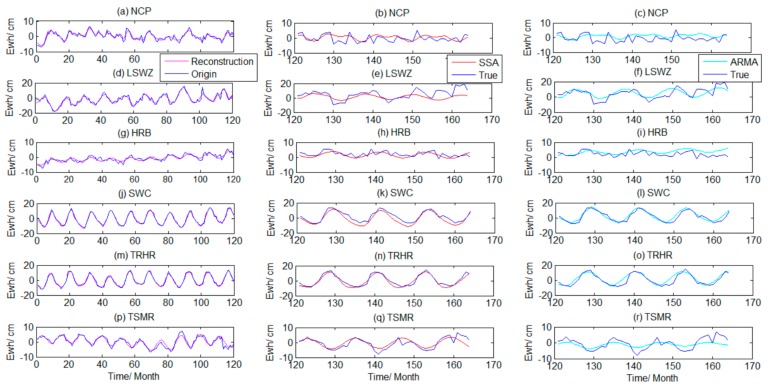
Singular spectrum analysis (SSA) reconstruction and the prediction time series from SSA and auto-regressive and moving average model (ARMA).

January 2013 was considered to be the 121st month, August 2016 the 164th, and so on. Based on the principle of the SSA prediction method, the regional TWSA in the last 44 months were predicted, as shown in Figure 5.

Figure 5 shows that the TWSA from SSA reconstruction in the six regions in the previous period agreed well with the actual GRACE sequence. The TWSA predicted by SSA in the late period of months 121–164 (Figure 5b NCP–5(q) TSMR) agreed well with the true value series, and the TWS predictions were closer to the truth sequence in the SWC, TRHR, and TSMR regions. The TWSA predictions in the NCP, LSWZ, and HRB deviated considerably from the true value in partial periods. The TWSA in these areas could be relatively complex, the periodic signal strength weak, and the ability of SSA to predict restricted. In month 160 of the end period, the SSA prediction timing and the true value had different degrees of deviation, and the prediction results in the LSWZ deviated the most.

To quantitatively assess the SSA predictive ability, the duration of months 121–132 was designated as short-term, months 121–144 as mid-short term, months 121–156 as medium-term, and months 121–164 as long-term. Based on the SSA prediction principle, the prediction results on four scales were compared with true value signals, and then we evaluated the accuracy. In this study, we calculated *R*, the proximity NSE, and the RMSE (cm), as shown in Table 3.

Table 3 shows that, in the short-term, compared with other regions, the SSA prediction accuracy of TWSA in the SWC and TRHR regions was the highest, the correlation coefficient with the true value was above 0.95, the NSE was 0.95, and the RMSE was smaller than 2 cm. The prediction accuracy in the TSMR area was also high; the *R*, NSE, and RMSE were 0.94, 0.88, and 1.07 cm, respectively. The *R* of the prediction time in LSWZ was 0.87, NSE was 0.61, and RMSE was 4.27 cm. The prediction accuracy of the HRB in terms of *R*, NSE, and RMSE was 0.67, 0.35, and 2.16 cm, respectively. The prediction accuracy in the NCP was lower than in the other five regions. We found that, as the prediction duration increased, the accuracy of SSA prediction in the six regions gradually decreased. However, the prediction accuracy for the SWC and the TRHR areas was still high in the long-term, followed by the TSMR area because the SWC and TRHR were nearly sinusoidal. This showed that SSA could effectively predict the GRACE signals with an obvious period and trend. For the regions with poor periodic signal strength (e.g., NCP), the prediction results had large uncertainty in long-term prediction, which may be related to the complex TWSA in the region itself. Human factors also had a strong impact, but the results still showed good agreement in the partial periods (e.g., months 120–129).

#### 4.2.3. Comparison with ARMA Forecast

**Figure 6:**
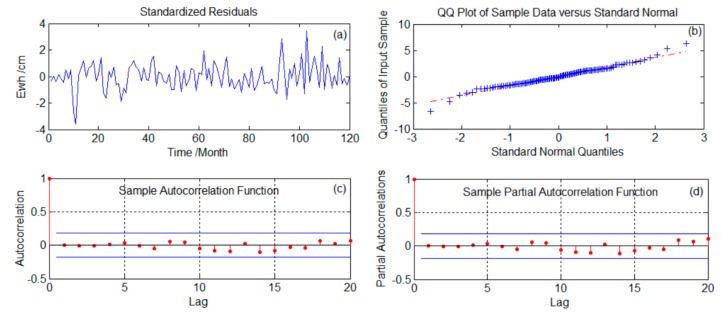
Test results of the ARMA model established in this study.

To highlight the prediction advantages of the SSA method, we compared it with ARMA. First, the GRACE time series were smoothed and zero-average processed. The AIC was used to determine the order, and after the model was fixed, the moment estimation method was used to estimate the unknown parameters of the model. Then, the validity of the model was tested. The test criteria were that the residual signal after extracting the fitted sample signal should be similar to white noise, and that the autocorrelation function and partial correlation function of the residual sequence should have obvious truncation. The test results for the constructed model in this paper are shown in Figure 6a NCP–6(d) TSMR. We used the available and different ARMA(p,q) model in different regions; *p*- and q-values were tested and verified. The ARMA(p,q) model used in the NCP was ARMA(9,10), ARMA(9,7) was used in LSWZ, ARMA(9,2) in HRB, ARMA(8,6) in SWC, ARMA(6,7) in TRHR, and ARMA(2,3) in TSMR.

An applicability test was conducted on the established ARMA model. The Ljung–Box–Pierce Q statistic was calculated for the 1st–10th-order lag of the model. The Q statistic of the model was smaller than the $\chi^{2}$ statistic, with a significance level of 0.05. Therefore, we found that the order and parameters of the ARMA model, established here, were suitable and could be used for prediction.

From the ARMA prediction results in Figure 5c NCP–5(r) TSMR (longitudinal), the ARMA model could also predict the overall TWSA, especially in regions with strong periodic signals. The prediction results in Figure 5l,o,r were better. However, the predicted sequence deviated more from the true value than the SSA result. The results in Table 3 highlight the accuracy of ARMA prediction for different durations in the six regions. In addition to the long-term scale for LSWZ, the *R* and NSE of ARMA prediction results were lower than those of the SSA model, and RMSE was larger. This shows that the SSA method better predicted the GRACE signal than the ARMA method.

In the follow-up study, the SSA method was used to predict the TWSA in the six regions from September 2016 to December 2017. To verify the reliability of the prediction results, the results were compared with Mascon RL06 and GLDAS results.

### 4.3. Prediction of Regional TWSA

#### 4.3.1. TWSA Forecast

**Figure 7:**
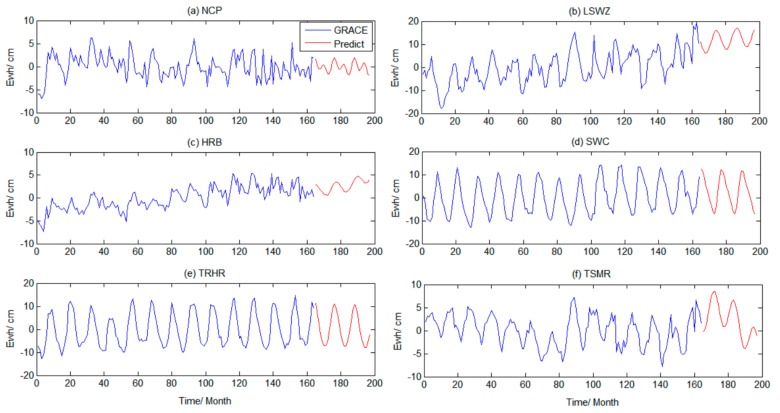
Time series of GRACE inversion results and SSA prediction results.

Based on the previous experimental results, we found that the prediction accuracy of SSA in the short, mid-short, and mid-term scales was relatively high. To bridge the gap between GRACE and GRACE-FO data, and to compare our prediction results with the latest GRACE-FO data (from June 2018 to May 2019) and the GLDAS model, the TWSA in the six regions from September 2016 to May 2019 were predicted using SSA from months 165 to 197, as shown in Figure 7.

Figure 7 shows that the regional TWSA predicted by SSA better continued the time series characteristics of the existing time interval (1th–164th month) in their respective regions. The variation in TWS in different regions was different and had obvious regional characteristics. Next, the Mascon RL06 and GLDAS models were used for verification analysis.

#### 4.3.2. Comparative Analysis with Mascon RL06 and GLDAS

**Figure 8:**
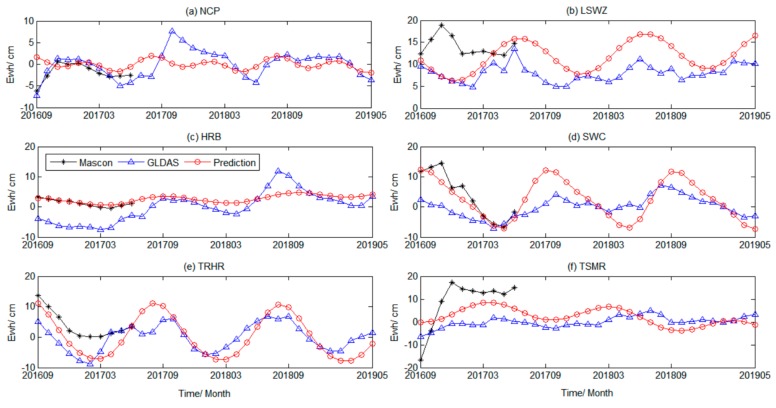
Comparison of GRACE TWS predicted by SSA with Mascon and GLDAS results. The red curve represents the prediction time series, the black curve represents the Mascon time series, and the blue curve represents the GLDAS time series.

To verify the reliability of the prediction results, we calculated the temporal change in the Mascon RL06, GLDAS-Noah model, and prediction results from September 2016 to May 2019. The three results are shown in Figure 8.

Figure 8a shows that, from September 2016 to May 2019, the predicted changes in TWS in the NCP were relatively stable, rising slightly in June 2017 and slowly decreasing in October. Fluctuations in 2018 were relatively weak; water storage in 2019 was trending downwards. The overall trend in predicted TWS in LSWZ increased year after year. Water storage peaked in June 2017 and June 2018, and was in a trough in December 2016, 2017, and 2018. The predicted changes in water storage in HRB fluctuated substantially in the small range of the zero axis, and the periodic signal characteristics were relatively weak. The annual variation in water storage in SWC was about 10 cm, and the water storage was the highest in May of each year. The annual water storage was the largest in September. The water storage in TRHR also showed obvious cyclical changes. TWS suffered the largest losses in March each year; the annual water storage increased by nearly 10 cm in August. The water storage in TSMR began to gradually decrease after the upward trend before March 2017. By September 2017, the water storage no longer decreased and began to increase each month. After March 2018, the water storage showed a relatively obvious downward trend. In September, it decreased to its minimum and then slowly rose. Figure 8 shows that the TWSA predicted by SSA were closer to the GLDAS results in terms of overall trends. The Mascon RL06 period ranged from September 2016 to June 2017. During this period, deviations occurred; except for individual months, the overall SSA forecast time series was also consistent with the Mascon results. 

#### 4.3.3. Comparison with GRACE-FO Data Test

To more effectively verify the effectiveness of the SSA iterative prediction method, the GRACE-FO data results were compared with our findings.

**Figure 9:**
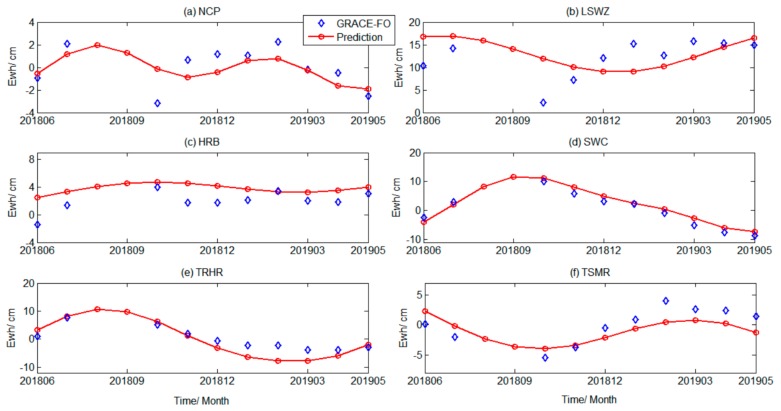
Comparison of predicted GRACE TWS changes with GRACE-FO results. The blue scatters represent the GRACE-FO results and the red curve expresses the change of TWS predicted within the 10 months.

The GRACE-FO data period ranged from June 2018 to May 2019, with a total of 10 months of data, and August and September 2018 data were missing. Since the published data period is relatively short, the interpolation of missing data was not considered here. According to the same process as GRACE, the water storage of the study areas were obtained, and the leakage errors of the study areas were corrected using the same scale factors as before (Figure 9). 

Figure 9 shows that the GRACE water storage predicted by the SSA iterative method displayed certain periodicity and trend characteristics during the period from June 2018 to May 2019, and the temporal variation and the overall trend were consistent with the results derived from GRACE-FO, especially the predicted water storage changes in Southwest China, the TRHR area, and the TSMR area. This indicates that the SSA method could better predict the regional TWSA monitored by GRACE within a certain time scale. If precipitation, temperature, wind speed, human factors, etc., were used as inputs to constrain the prediction results, the prediction accuracy may be further improved. This was explained in the discussion section.

## 5. Discussion

### 5.1. Applicability Test of the SSA+ARMA Model

Since the idea of SSA prediction is to reconstruct the period and trend signals, residual sequences remain after SSA reconstruction. We wanted to know if the ARMA model could improve the prediction accuracy if used to model the residual sequence. Therefore, we also conducted related experiments using the SSA+ARMA model to predict the TWSA in the six regions, and found that the accuracy of the prediction results was slightly lower than that of the SSA method, but still better than the accuracy of the single ARMA model. This indicates that the residual components remaining after extracting the PCs using the SSA method were not suitable for ARMA optimal modeling. Therefore, trying to explore a method suitable for SSA residual component modeling to highlight the detail signal may contribute to improving prediction accuracy.

### 5.2. Prediction Uncertainty

The SSA prediction results better reflect the trend and the periodic item signals, but the actual TWSA could be affected by extreme climate and human factors, and the TWSA produced different degrees of mutation or spike. Lacking meteorological, human, and other factors as constraint data, SSA prediction results may have some uncertainty. If meteorological data, such as precipitation, temperature, humidity, wind speed, evaporation, etc., are used as input parameters, the accuracy of the prediction results may improve, which coincides well with the TWSA characteristics of specific areas. The related content will also be improved in our further research. In our follow-up study, the prediction period will be extended to the GRACE-FO data start period to link the gap between the gravity satellite data of two generations.

### 5.3. Inconsistency of GRACE TWS Relative to Mascon and GLDAS Predictions

A difference exists in GRACE TWS relative to Mascon and GLDAS predictions because the GRACE and Mascon results were obtained by two different data post-processing methods, producing some differences in spatial resolution and precision. The GRACE results had larger amplitudes than the GLDAS hydrological model, mainly because that only changes in soil water and snow cover were included in GLDAS, whereas GRACE reflected changes in total terrestrial water storage and possible physical migration of solid earth [49]. Therefore, the three data sources had the same characteristics and some differences, and the GRACE predictions mainly reflected periodic and trend changes and did not consider external data constraints. The above factors possibly increased their inconsistency.

## 6. Conclusions

In this paper, considering the data gap between GRACE and GRACE-FO, the SSA method was proposed to predict the TWSA derived from GRACE in a finite period. The time series of TWS from January 2003 to August 2016 was obtained by GRACE post-processing, and we compared it with the Mascon results of CSR, GFZ, and JPL to verify the reliability of GRACE inversion results so that we could conduct SSA prediction research. Our research results indicated:

(1) The variations in TWS derived from GRACE in the limited time period had good consistency with the Mascon products from the three institutions, and the highest degree of coincidence with Mascon RL06 indicated that the GRACE inversion results were reliable.

(2) The signal prediction accuracy of the TRHR and SWC regions was relatively high, which may be related to the strong periodic signals of the two regions. As forecast duration increased, the prediction accuracy of the six regions gradually decreased.

(3) The regional TWSA prediction accuracy using the ARMA model was lower than that of the SSA method in different experimental areas and most time scales.

(4) The regional TWSA predicted by the SSA method had obvious periodic and trend characteristics, which were basically consistent with the GLDAS model. In addition to the large difference in the LSWZ regions, the prediction time series of the other regions were also in good agreement with Mascon RL06. The predicted TWSA agreed with GRACE-FO results overall. However, the detailed signal prediction by SSA method cannot be highlighted effectively. 

The method proposed in this paper provides an important reference for the continuous monitoring of water resource changes, which has a strong scientific significance.

## Figures and Tables

**Table 1:** Filtering scale factor calculated using the single-scale factor method in the six study regions in China.

Region Name	NCP	SWC	TRHR	TSMR	HRB	LSWZ
Scale factor	1.28	1.16	1.06	0.97	0.76	1.22

**Table 2:** Accuracy indicators of regional TWSA compared to Mascon results. NSE: Nash–Sutcliffe efficiency; *R*: correlation coefficient.

*R*/NSE	JPL RL05	CSR RL05	GFZ RL05	JPL RL06
NCP	0.42/0.01	0.48/0.08	0.52/0.11	0.58/0.18
SWC	0.95/0.69	0.95/0.76	0.94/0.74	0.95/0.90
TRHR	0.97/0.92	0.98/0.96	0.97/0.94	0.97/0.95
TSMR	−0.39/−0.15	−0.51/−0.29	−0.49/−0.27	0.88/0.58
HRB	0.40/0.08	0.72/0.25	0.71/0.41	0.92/0.74
LSWZ	0.87/0.66	0.90/0.72	0.88/0.67	0.93/0.87

**Table 3:** Accuracy evaluation of regional TWSA prediction from January 2013 to August 2016.

Area	Method	Short-Term	Mid-Short Term	Medium-Term	Long-Term
NCP	SSA	0.51/0.26/2.37	0.45/0.20/2.38	0.36/0.08/2.34	0.34/2.27/0.03
	ARMA	0.36/0.12/2.58	0.30/0.07/2.56	0.27/0.03/2.40	0.26/0.02/2.29
LSWZ	SSA	0.87/0.61/4.27	0.85/0.59/3.89	0.70/0.44/4.41	0.58/0.31/5.55
	ARMA	0.73/0.54/4.63	0.76/0.57/3.98	0.63/0.39/4.61	0.65/0.42/5.11
HRB	SSA	0.67/0.35/2.16	0.65/0.32/2.26	0.61/0.24/2.38	0.56/0.09/2.54
	ARMA	0.59/0.32/1.39	0.53/0.23/1.51	0.43/0.05/1.66	0.28/‒0.21/1.812
SWC	SSA	0.98/0.95/1.83	0.96/0.92/2.22	0.96/0.91/2.29	0.96/0.90/2.27
	ARMA	0.97/0.94/2.02	0.95/0.90/2.27	0.94/0.88/2.40	0.93/0.86/2.57
TRHR	SSA	0.97/0.95/1.73	0.96/0.92/2.10	0.95/0.91/2.30	0.95/0.90/2.37
	ARMA	0.94/0.89/2.55	0.91/0.84/3.08	0.90/0.81/3.33	0.91/0.82/3.14
TSMR	SSA	0.94/0.88/1.07	0.91/0.81/1.40	0.88/0.77/1.48	0.78/0.60/2.21
	ARMA	0.93/0.68/1.72	0.89/0.54/2.18	0.82/0.49/2.18	0.78/0.40/2.70
